# Supplementary material for: Exploring the potential role of ENPP2 in polycystic ovary syndrome and endometrial cancer through bioinformatic analysis
Source: PeerJ. 2024 Dec 20;12:e18666. doi: 10.7717/peerj.18666 (PMC11665432; doi:10.7717/peerj.18666)
Supplement: Supplemental Information 2 — Primer sequences of ENPP2 for reverse transcription-quantitative polymerase chain reaction. AR-siRNA and pcDNA3.1-ENPP2 Primer sequences. [file peerj-12-18666-s002.docx]

Table S1 AR-siRNA and pcDNA3.1-ENPP2 Primer sequences

| **Gene** | **Forward (5′-3′)** | Reverse (5′-3′) |
| --- | --- | --- |
| NC siRNA | 5ʹ-UUCUCCGAACGUGUCACGUUU-3ʹ | 5ʹ-ACGUGACAGGUUCGGAGAAUU-3' |
| AR siRNA-1 | 5ʹ- CGAGUUUGCAGAGAGGUAACU-3ʹ | 5ʹ- UUACCUCUCUGCAAACUCGGG -3' |
| AR siRNA-2 | 5ʹ- CGGGAAGUUUAGAGAGCUAAG-3ʹ | 5ʹ- UAGCUCUCUAAACUUCCCGUG -3' |
| pcDNA3.1-ENPP2 | 5ʹ-TACCGAGCTCGGATCCATGGCAAGGAGGAGCTCGTTC-3ʹ | 5ʹ-GATATCTGCAGAATTCTTAAATCTCGCTCTCATATG-3ʹ |
