# Supplementary figures and images for: Exploring the potential role of ENPP2 in polycystic ovary syndrome and endometrial cancer through bioinformatic analysis
Source: PeerJ. 2024 Dec 20;12:e18666. doi: 10.7717/peerj.18666 (PMC11665432; doi:10.7717/peerj.18666)

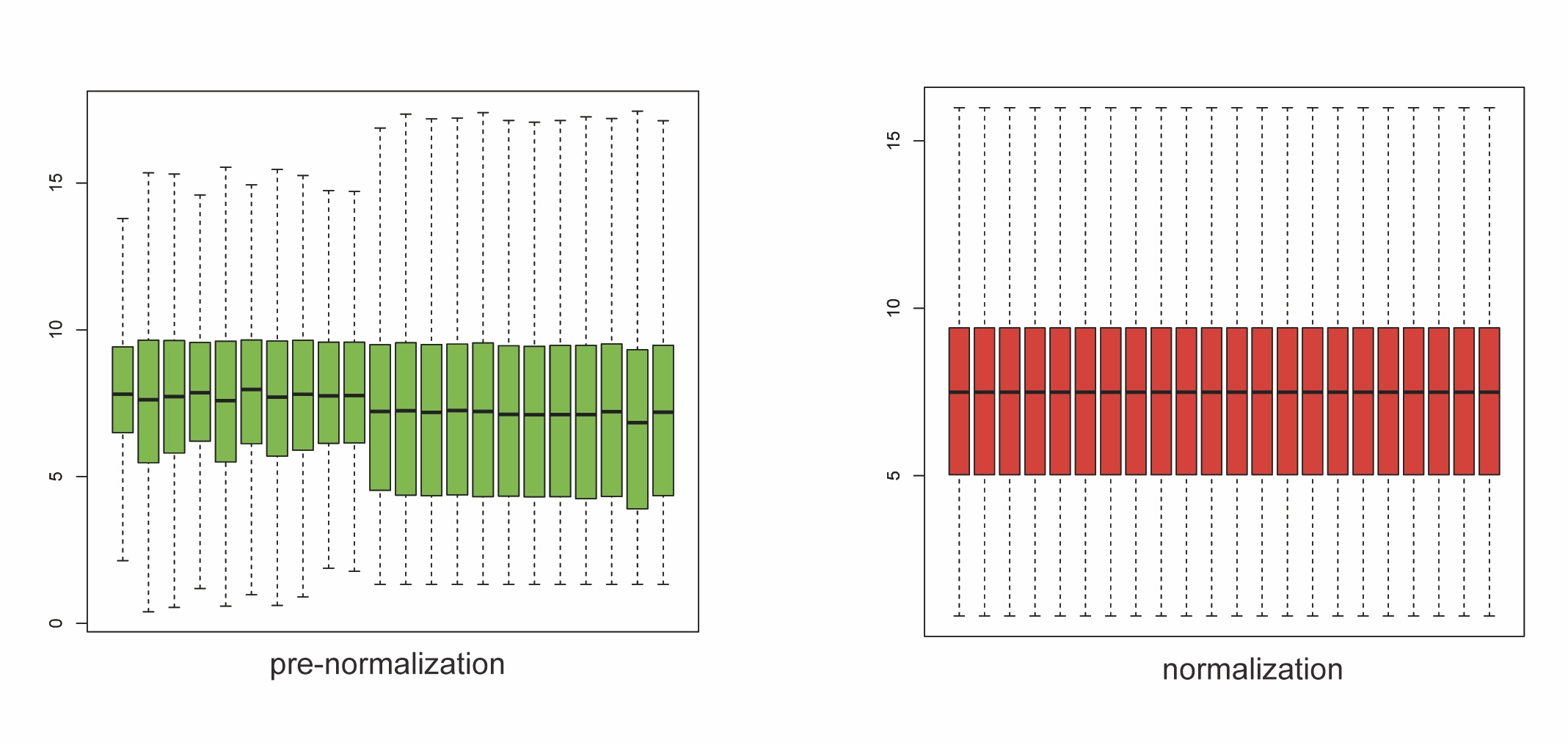

Supplement: Supplemental Information 3 [file peerj-12-18666-s003.jpg]

A

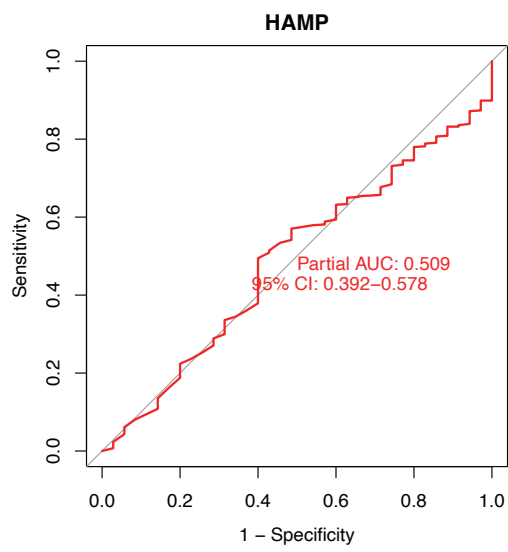

B

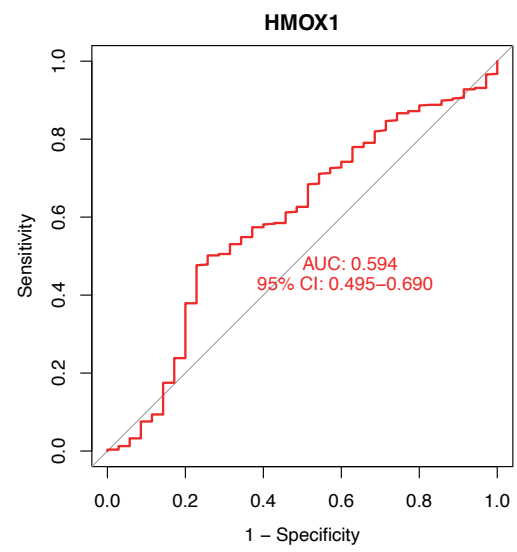

Supplement: Supplemental Information 4 — Verified the accuracy of HAMP and HMOX1 in TCGA-UCEC cohort by receiver operating characteristic (ROC) curves. [file peerj-12-18666-s004.pdf]

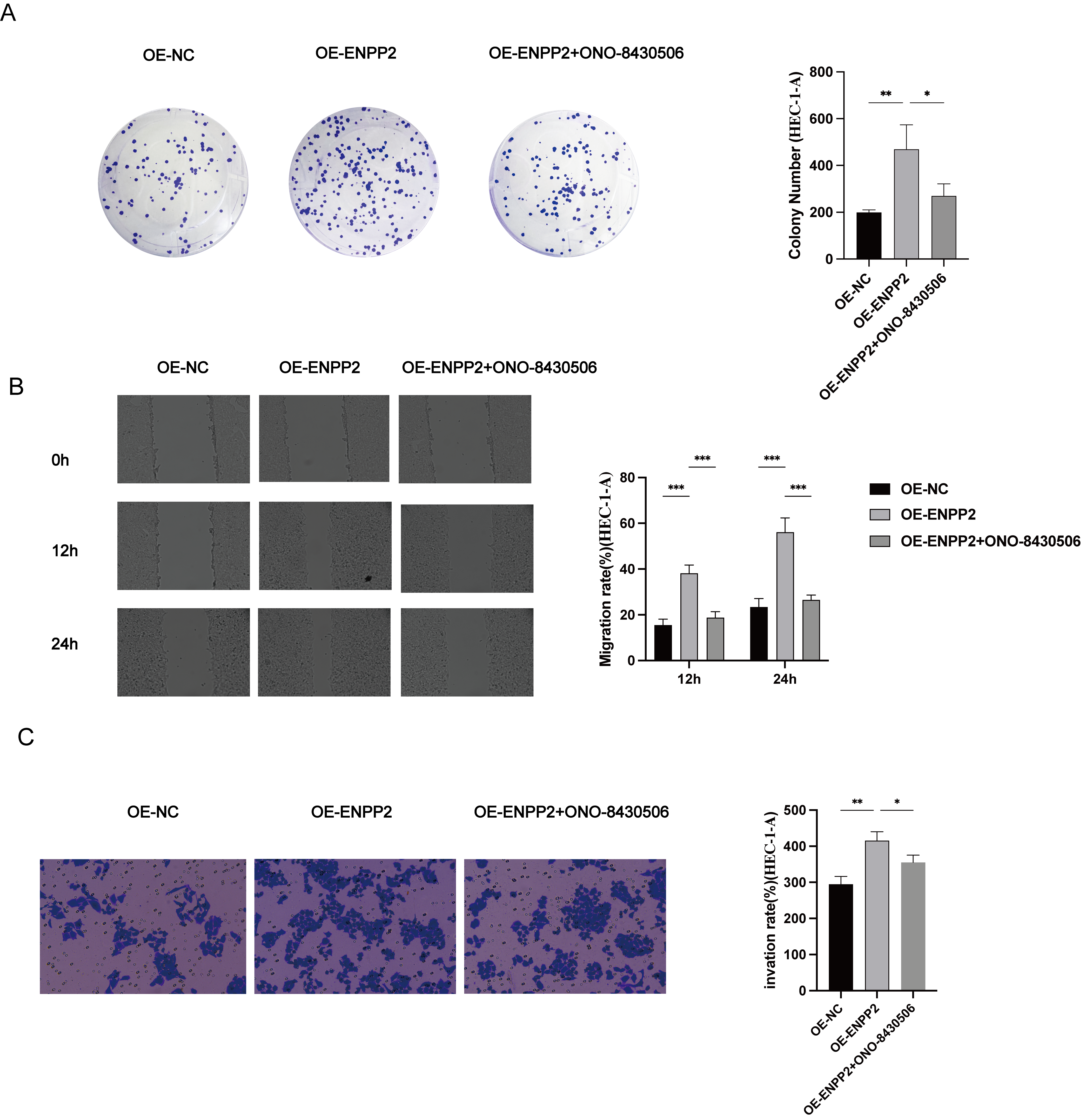

Supplement: Supplemental Information 5 — (a) The mRNA levels in overexpression normal control (OE-NC) and ENPP2 overexpression (OE-ENPP2) HEC-1-A cells. (b) OE-ENPP2 HEC-1-A cells were cultured for 10 days or until more than 50 monoclonal cells, and 10nM ONO-8430506 was added to inhibit ENPP2 expression. (c) Migration rates of HEC-1-A cells at 12 h and 24 h. (d) Transwell invasion rates of HEC-1-A cells at 24 h. *p<0.05, **p<0.01, ***p<0.001 [file peerj-12-18666-s005.png]

DHT-HEC-1-A


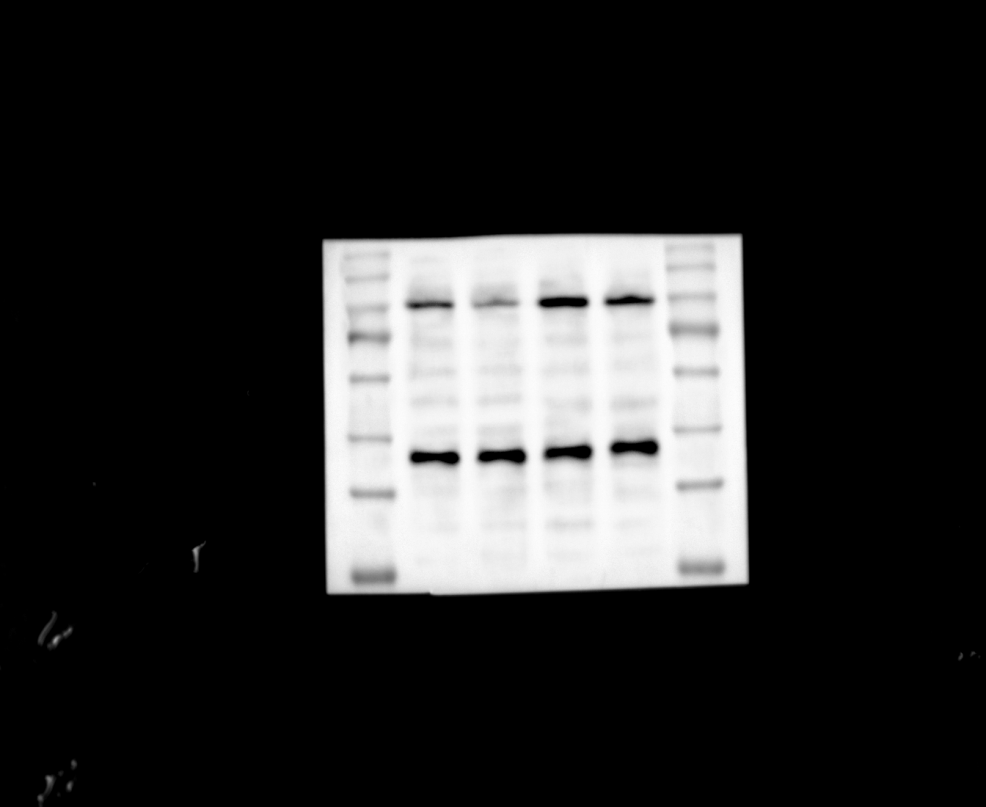


DHT-ISK


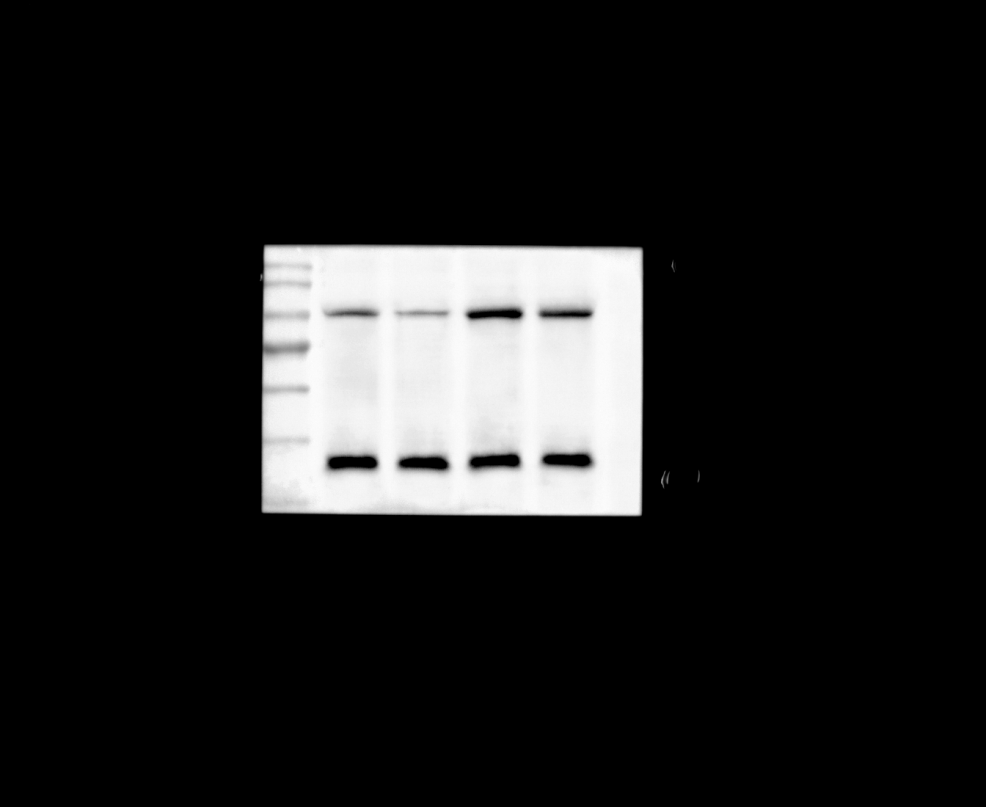


CGs


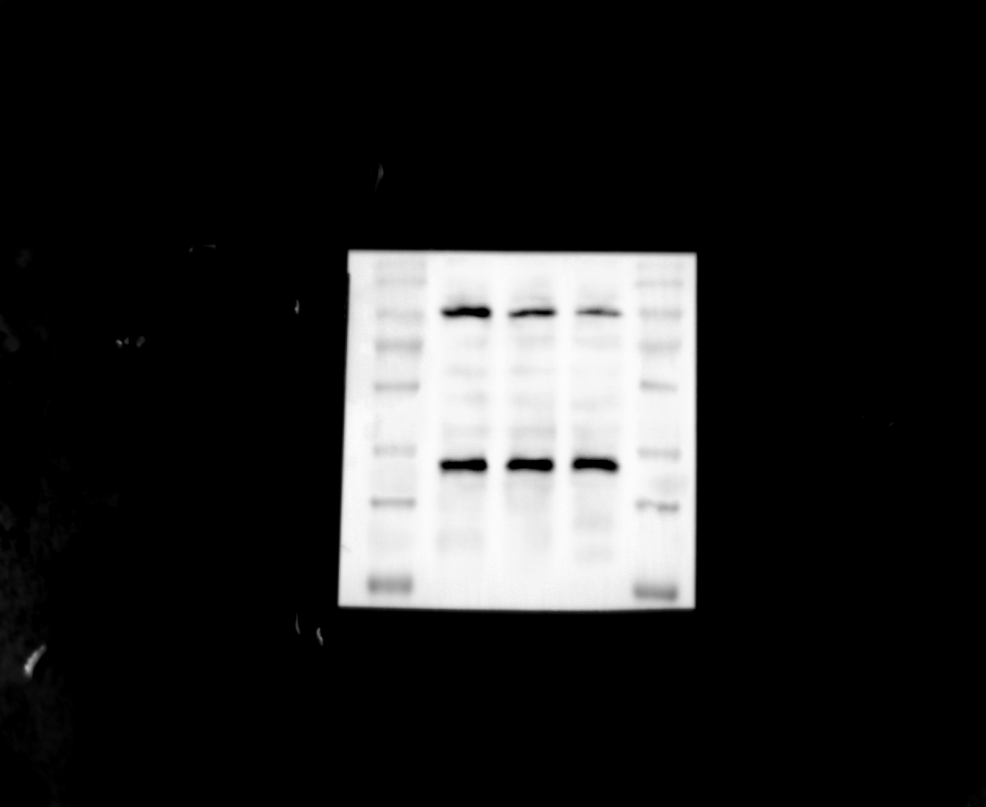


endometrium


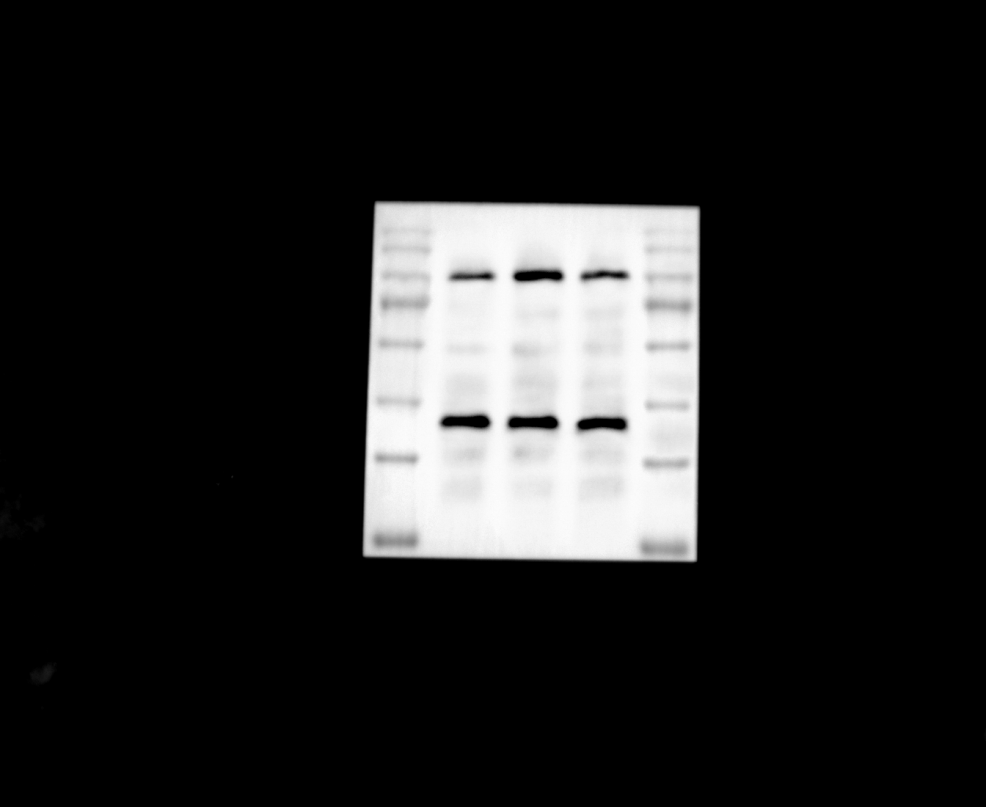

Supplement: Supplemental Information 6 [file peerj-12-18666-s006.docx]
