# Supplementary material for: Exploring the potential role of ENPP2 in polycystic ovary syndrome and endometrial cancer through bioinformatic analysis
Source: PeerJ. 2024 Dec 20;12:e18666. doi: 10.7717/peerj.18666 (PMC11665432; doi:10.7717/peerj.18666)
Supplement: Supplemental Information 1 [file peerj-12-18666-s001.zip › RT-qPCR data/RT-qpcr.doc]

Endometrium

| **group** | **Target** | **Mean Ct** | beta-actin_**CT** | **⊿Ct** | **⊿Ct Mean** | **⊿⊿Ct** | 2-⊿⊿Ct |
| --- | --- | --- | --- | --- | --- | --- | --- |
| control | ENPP2 | 25.53 | 14.81 | 10.73 | 9.62 | 1.11 | 0.46 |
| control | ENPP2 | 25.21 | 15.03 | 10.18 | 9.62 | 0.56 | 0.68 |
| control | ENPP2 | 25.54 | 15.23 | 10.32 | 9.62 | 0.70 | 0.62 |
| control | ENPP2 | 25.06 | 15.33 | 9.72 | 9.62 | 0.10 | 0.93 |
| HA-PCOS | ENPP2 | 24.19 | 15.52 | 8.67 | 9.62 | -0.95 | 1.93 |
| HA-PCOS | ENPP2 | 24.35 | 15.32 | 9.03 | 9.62 | -0.59 | 1.51 |
| HA-PCOS | ENPP2 | 24.08 | 14.93 | 9.15 | 9.62 | -0.47 | 1.38 |
| HA-PCOS | ENPP2 | 24.18 | 15.53 | 8.65 | 9.62 | -0.97 | 1.96 |
| NA-PCOS | ENPP2 | 25.12 | 15.50 | 9.62 | 9.62 | 0.00 | 1.00 |
| NA-PCOS | ENPP2 | 25.12 | 15.80 | 9.32 | 9.62 | -0.30 | 1.23 |
| NA-PCOS | ENPP2 | 24.40 | 15.04 | 9.36 | 9.62 | -0.26 | 1.20 |
| NA-PCOS | ENPP2 | 23.88 | 14.55 | 9.34 | 9.62 | -0.28 | 1.22 |

Granule cells

| **group** | **Target** | **Mean Ct** | beta-actin_**CT** | **⊿Ct** | **⊿⊿Ct** | **2-**⊿⊿Ct |
| --- | --- | --- | --- | --- | --- | --- |
| control | ENPP2 | 22.20 | 16.12 | 6.09 | -1.97 | 3.91 |
| control | ENPP2 | 22.24 | 15.32 | 6.92 | -1.13 | 2.19 |
| control | ENPP2 | 22.04 | 15.53 | 6.51 | -1.55 | 2.92 |
| control | ENPP2 | 22.21 | 15.04 | 7.17 | -0.88 | 1.84 |
| HA-PCOS | ENPP2 | 23.76 | 14.93 | 8.84 | 0.78 | 0.58 |
| HA-PCOS | ENPP2 | 23.82 | 15.80 | 8.02 | -0.03 | 1.02 |
| HA-PCOS | ENPP2 | 23.03 | 14.81 | 8.22 | 0.17 | 0.89 |
| HA-PCOS | ENPP2 | 23.37 | 15.03 | 8.34 | 0.28 | 0.82 |
| NA-PCOS | ENPP2 | 23.56 | 15.50 | 8.05 | 0.00 | 1.00 |
| NA-PCOS | ENPP2 | 23.74 | 15.23 | 8.51 | 0.46 | 0.73 |
| NA-PCOS | ENPP2 | 24.42 | 15.33 | 9.09 | 1.03 | 0.49 |
| NA-PCOS | ENPP2 | 22.48 | 14.55 | 7.93 | -0.12 | 1.09 |

ISK

| group | beta-actin_CT | Target | ΔCt | —ΔΔCt | 2-⊿⊿Ct |
| --- | --- | --- | --- | --- | --- |
| si-con (AR) | 18.97 | 24.94 | 5.97 | -0.18 | 0.88 |
|  | 19.26 | 24.97 | 5.71 | 0.08 | 1.06 |
|  | 19 | 24.7 | 5.7 | 0.09 | 1.07 |
| si-1 (AR) | 18.97 | 26.58 | 7.61 | -1.82 | 0.28 |
|  | 19.26 | 26.51 | 7.25 | -1.46 | 0.36 |
|  | 19 | 26.47 | 7.47 | -1.68 | 0.31 |
| si-2 (AR) | 18.97 | 26.41 | 7.44 | -1.65 | 0.32 |
|  | 19.26 | 26.4 | 7.14 | -1.35 | 0.39 |
|  | 19 | 26.42 | 7.42 | -1.63 | 0.32 |
| OE-con | 18.97 | 24.85 | 5.88 | -0.09 | 0.94 |
|  | 19.26 | 24.76 | 5.5 | 0.29 | 1.23 |
|  | 19 | 24.82 | 5.82 | -0.03 | 0.98 |
| OE-ENPP2 | 18.97 | 23.38 | 4.41 | 1.38 | 2.61 |
|  | 19.26 | 23.67 | 4.41 | 1.38 | 2.61 |
|  | 19 | 23.73 | 4.73 | 1.06 | 2.09 |

HEC-1-A

| group | beta-actin_CT | Target | ΔCt | —ΔΔCt | 2-⊿⊿Ct |
| --- | --- | --- | --- | --- | --- |
| si-con (AR) | 19.1 | 24.92 | 5.82 | -0.13 | 0.91 |
|  | 19.76 | 25.22 | 5.46 | 0.23 | 1.17 |
|  | 19 | 24.78 | 5.78 | -0.09 | 0.94 |
| si-1 (AR) | 19.1 | 26.53 | 7.43 | -1.74 | 0.30 |
|  | 19.76 | 26.61 | 6.85 | -1.16 | 0.45 |
|  | 19 | 26.33 | 7.33 | -1.64 | 0.32 |
| si-2 (AR) | 19.1 | 26.39 | 7.29 | -1.60 | 0.33 |
|  | 19.76 | 26.6 | 6.84 | -1.15 | 0.45 |
|  | 19 | 26.38 | 7.38 | -1.69 | 0.31 |
| OE-con | 19.1 | 24.44 | 5.34 | 0.35 | 1.27 |
|  | 19.76 | 24.74 | 4.98 | 0.71 | 1.63 |
|  | 19 | 24.72 | 5.72 | -0.03 | 0.98 |
| OE-ENPP2 | 19.1 | 23.37 | 4.27 | 1.42 | 2.67 |
|  | 19.76 | 23.56 | 3.8 | 1.89 | 3.70 |
|  | 19 | 23.21 | 4.21 | 1.48 | 2.78 |

| gene | group | beta-actin_CT | Target | ΔCt | —ΔΔCt | 2-⊿⊿Ct |
| --- | --- | --- | --- | --- | --- | --- |
| AR | con | 18.9 | 24.37 | 5.47 | 0.10 | 1.07 |
|  |  | 19.23 | 24.78 | 5.55 | 0.02 | 1.02 |
|  |  | 19.1 | 24.8 | 5.7 | -0.13 | 0.92 |
|  | OE-ENPP2 | 18.9 | 23.81 | 4.91 | 0.66 | 1.58 |
|  |  | 19.23 | 24.1 | 4.87 | 0.70 | 1.63 |
|  |  | 19.1 | 24.22 | 5.12 | 0.45 | 1.37 |
|  | OE-ENPP2+  ONO-8430506 | 18.9 | 24.1 | 5.2 | 0.37 | 1.30 |
|  |  | 19.23 | 24.73 | 5.5 | 0.07 | 1.05 |
|  |  | 19.1 | 24.5 | 5.4 | 0.17 | 1.13 |
| CYP19A1 | con | 18.9 | 24.31 | 5.41 | 0.16 | 1.12 |
|  |  | 19.23 | 25 | 5.77 | -0.20 | 0.87 |
|  |  | 19.1 | 24.77 | 5.67 | -0.10 | 0.94 |
|  | OE-ENPP2 | 18.9 | 23.7 | 4.8 | 0.77 | 1.71 |
|  |  | 19.23 | 23.88 | 4.65 | 0.92 | 1.90 |
|  |  | 19.1 | 23.69 | 4.59 | 0.98 | 1.98 |
|  | OE-ENPP2+  ONO-8430506 | 18.9 | 23.73 | 4.83 | 0.74 | 1.67 |
|  |  | 19.23 | 24.35 | 5.12 | 0.45 | 1.37 |
|  |  | 19.1 | 24.21 | 5.11 | 0.46 | 1.38 |
